# Quantitative reliability assessment of brain MRI volumetric measurements in type II GM1 gangliosidosis patients

**DOI:** 10.3389/fnimg.2024.1410848

**Published:** 2024-09-13

**Authors:** Christopher Zoppo, Josephine Kolstad, Jean Johnston, Precilla D'Souza, Anna Luisa Kühn, Zeynep Vardar, Ahmet Peker, Clifford Lindsay, Zubir S. Rentiya, Robert King, Heather Gray-Edwards, Behroze Vachha, Maria T. Acosta, Cynthia J. Tifft, Mohammed Salman Shazeeb

**Affiliations:** ^1^Image Processing and Analysis Core (iPAC), Department of Radiology, University of Massachusetts Chan Medical School, Worcester, MA, United States; ^2^National Human Genome Research Institute, National Institutes of Health, Bethesda, MD, United States; ^3^Horae Gene Therapy Center, University of Massachusetts Chan Medical School, Worcester, MA, United States; ^4^Division of Neuroradiology, Department of Radiology, University of Massachusetts Chan Medical School, Worcester, MA, United States

**Keywords:** GM1 gangliosidosis, inter/intra-rater reliability, brain MRI, volumetrics, segmentation

## Abstract

**Purpose:**

GM1-gangliosidosis (GM1) leads to extensive neurodegenerative changes and atrophy that precludes the use of automated MRI segmentation techniques for generating brain volumetrics. We developed a standardized segmentation protocol for brain MRIs of patients with type II GM1 and then assessed the inter- and intra-rater reliability of this methodology. The volumetric data may be used as a biomarker of disease burden and progression, and standardized methodology may support research into the natural history of the disease which is currently lacking in the literature.

**Approach:**

Twenty-five brain MRIs were included in this study from 22 type II GM1 patients of which 8 were late-infantile subtype and 14 were juvenile subtype. The following structures were segmented by two rating teams on a slice-by-slice basis: whole brain, ventricles, cerebellum, lentiform nucleus, thalamus, corpus callosum, and caudate nucleus. The inter- and intra-rater reliability of the segmentation method was assessed with an intraclass correlation coefficient as well as Sorensen-Dice and Jaccard coefficients.

**Results:**

Based on the Sorensen-Dice and Jaccard coefficients, the inter- and intra-rater reliability of the segmentation method was significantly better for the juvenile patients compared to late-infantile (*p* < 0.01). In addition, the agreement between the two rater teams and within themselves can be considered good with all *p*-values < 0.05.

**Conclusions:**

The standardized segmentation approach described here has good inter- and intra-rater reliability and may provide greater accuracy and reproducibility for neuromorphological studies in this group of patients and help to further expand our understanding of the natural history of this disease.

## 1 Introduction

Gangliosidoses are heritable disorders of sphingomyelin breakdown due to deficient lysosomal enzymes in the brain and spinal cord (Bisel et al., 2014). The toxic accumulation of substrate (GM1- or GM2-gangliosides) leads to hypomyelination, neurodegeneration, and eventually death. While GM2-gangliosidosis affects only the central nervous system (CNS), GM1-gangliosidosis (GM1) affects the CNS as well as other organ systems (Regier et al., 2016a). There are three subtypes of GM1 defined by onset and severity: (1) infantile (type I), the most severe; (2) late-infantile and juvenile (type II); and (3) adult (type III), the least severe (King et al., 2020; Bley et al., 2011).

The neurodegeneration caused by this disease leads to many findings on MRI including hypomyelination, signal changes in the basal ganglia, and atrophy throughout the cerebellum and cerebrum (Steenweg et al., 2010; De Grandis et al., 2009; Nestrasil et al., 2018). These changes can be useful for qualitatively tracking the progression of the disease in individual patients (Nestrasil et al., 2018; Regier et al., 2016b). However, with the advent of innovative methods like gene therapy to treat patients with similar disorders (Flotte et al., 2022), the need to adequately quantify changes in the brain becomes more essential, especially in tracking disease progression and responses to treatment.

Brain volumetrics have been used as an acceptable biomarker to quantify pediatric brain structures (Yu et al., 2010; Hashempour et al., 2019; Phan et al., 2018), and also track brain structural changes in GM1 patients (Nestrasil et al., 2018; Regier et al., 2016b). Typically, the severity of GM1, especially in type I and type II pediatric patients, causes brain signal changes and affects the brain structure sizes compared to normal pediatric brains; hence adult or even pediatric brain atlases cannot be used to co-register GM1 patient brains for automated segmentation analysis. To our knowledge, no fully automated method exists to segment GM1 patient brain structures based on a natural history study brain atlas given the rarity of such patients. This necessitates some form of manual intervention to accurately segment and quantify brain structure volumes in GM1 patients.

In this paper, we describe a standardized protocol to segment brain structures facilitated by a manual process using software segmentation tools from a cohort of natural history type II GM1 patients. For clinical reliability, the brain structure segmentations need to be evaluated by multiple qualified reviewers and at multiple instances to ensure consistent and reproducible results. The goal of this study was to assess the inter- and intra-rater reliability of this standardized protocol for brain structure segmentations in type II GM1 patients.

## 2 Materials and methods

### 2.1 Subjects

Patients with a confirmed diagnosis of GM1 gangliosidosis were enrolled in National Institutes of Health (NIH) protocol 02-HG-0107: “The Natural History of Patients with Glycosphingolipid Storage Disorders.” Diagnosis was confirmed by biallelic mutations in the *GLB1* gene, a deficiency of beta-galactosidase enzyme levels in leukocytes, and a clinical presentation that fits the phenotype of a GM1 patient. Parents or legal guardians provided informed consent for patients' participation in the study. Twenty-five brain MRIs were included in this study from 22 type II GM1 patients of which 8 were late-infantile (5.9 ± 2.1 years) and 14 were juvenile (14.9 ± 6.2 years). The sample size was calculated based on intraclass correlation coefficient (ICC) guidelines that would provide a 95% confidence interval (CI) with a reasonable CI width for ICC values in the range of 0.7–0.9 (Bonett, 2002), which was the expected range from our preliminary measurements.

### 2.2 Imaging protocol

Brain MRI was performed on all patients under anesthesia using a Philips Achieva 3T system (Philips Healthcare, Best, the Netherlands) equipped with an 8-channel SENSE head coil (Philips Healthcare). The following scan sequences were acquired as part of the standard clinical MRI protocol without the use of intravenous contrast: 3D T1-weighted (T1W) fast field echo (FFE) imaging (TR/TE = 11/7 ms, slice thickness = 1 mm, flip angle = 6°, NEX = 2, FOV = 220 mm), axial T2-weighted imaging (TR/TE = 5,400/100 ms, slice thickness = 2 mm, flip angle = 90°, NEX = 1, FOV = 220 mm), axial fluid attenuated inversion recovery (FLAIR) imaging (TR/TE = 10,000/140 ms, inversion time = 2,650 ms, slice thickness = 5 mm, flip angle = 90°, NEX = 1, FOV = 220 mm), coronal short T1 inversion recovery (STIR) imaging (TR/TE = 4,000/14 ms, inversion time = 200 ms, slice thickness = 3 mm, flip angle = 90°, NEX = 1, FOV = 180 mm), and 3D magnetization-prepared rapid acquisition with gradient echo (MPRAGE) imaging (TR/TE = 8/4 ms, slice thickness = 1 mm, flip angle = 8°, NEX = 1, FOV = 220 mm).

### 2.3 Image analysis

**Figure 1:**
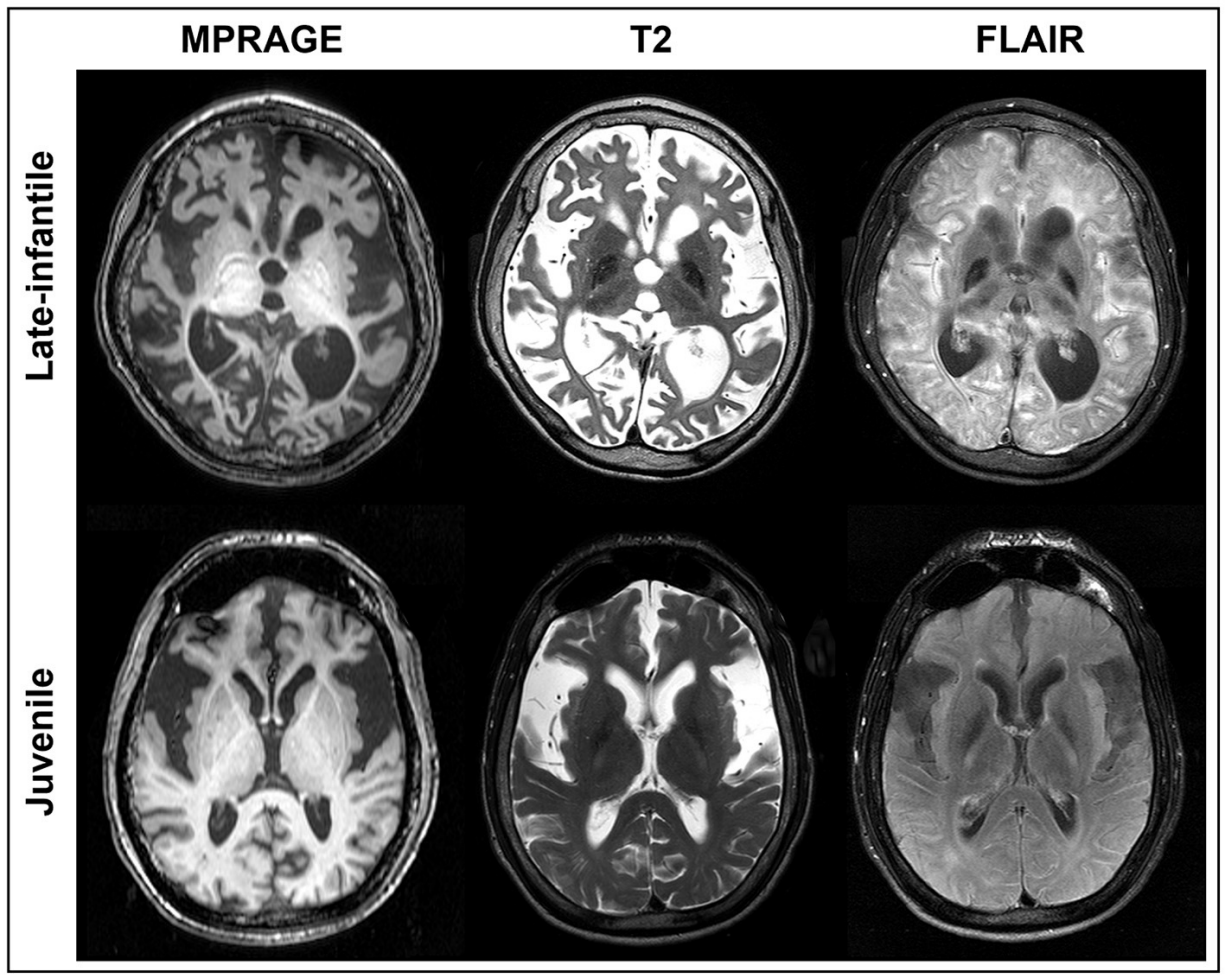
MRI of GM1 patients. Axial MPRAGE, T2, and FLAIR images are shown at the same level for a representative late-infantile **(top)** and juvenile **(bottom)** patient. While both cases demonstrate marked degeneration, the late-infantile patients were more severe, adding difficulty when generating regions of interest (ROIs). ROIs for each brain structure were determined based on the MPRAGE or 3D FFE images given the overall clarity of the structures.

Brain volumetric analysis was performed on either the 3D-FFE or 3D-MPRAGE scans, both of which provided sufficient T1W tissue contrast to visualize the brain structures of interest (Figure 1). Image corrections for Gibbs ringing artifacts and bias field inhomogeneity were not performed as their effects were minimal in the structures of interest for all patients. Two rater teams, each consisting of a neuroimaging expert and a board-certified radiologist with specialization in neuroradiology, independently used AMIRA (Thermo Fischer Scientific, Waltham, MA, USA) to perform brain structure segmentations on all 25 brain MRI scans. To increase the robustness of the analysis, the rater teams were blinded to the two patient groups (late-infantile and juvenile) being studied. The following structures, known to be implicated in the neurodegeneration seen with GM1, were segmented from all patients: whole brain (without ventricles), ventricles, cerebellum, lentiform nucleus, thalamus, corpus callosum, and caudate.

**Figure 2:**
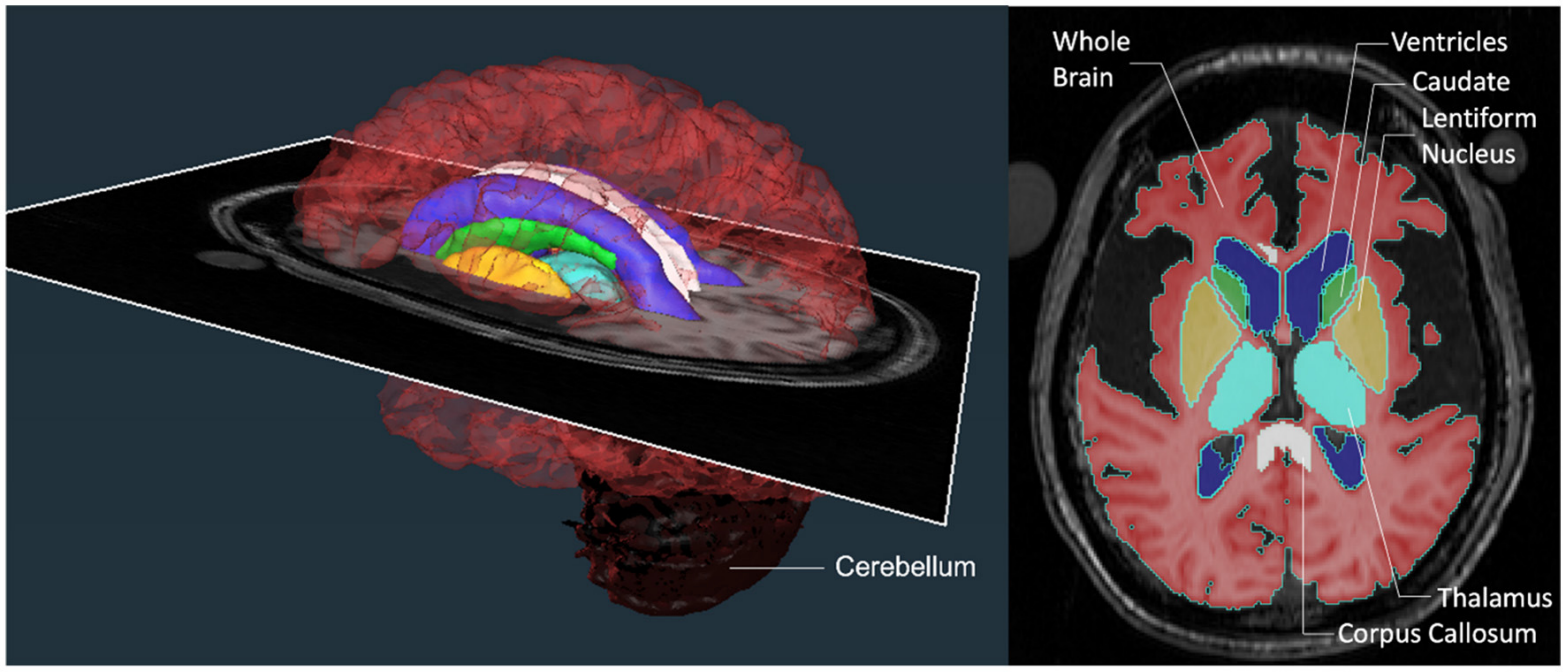
Brain region segmentations. A color-coded 3D rendering of a patient's brain with an axial MRI slice transecting it at the level of the image shown on the right. The axial slice on the right demonstrates the regions of interest (ROIs) that were segmented at each level on the scan to generate the 3D structures for comparison. The ROIs consisted of structures that were large (whole brain without ventricles, ventricles, and cerebellum) and small (lentiform nucleus, thalamus, corpus callosum, and caudate).

Segmentations were performed in the native space on a slice-by-slice basis by drawing a region of interest (ROI) around the structure either based on signal intensity threshold, manual demarcations, or both depending on the best-suited method for the respective structure. For each patient, the signal intensity threshold for each brain structure was selected to most effectively capture the structure of interest. The slice ROIs were then rendered into a 3D shape to estimate the volume of each structure (Figure 2). Each team repeated the segmentations using the same technique on all the scans at least 1 month after having completed the first set of measurements. The radiologist in each team independently verified all the structures for anatomical accuracy.

The following details for each structure were adopted to ensure consistent measurements and reduced variability between the teams and their repeated measurements.

#### 2.3.1 Whole brain

ROIs were automatically selected in the axial plane based on pixel intensity threshold of the brain matter without ventricles. The automatic selections were then manually edited for accuracy on every slice moving inferiorly until the pontomedullary junction where the segmentation was stopped. The ROIs were then viewed and confirmed in sagittal and coronal planes.

#### 2.3.2 Ventricles

In the axial plane, the pixel intensity threshold tool was used to automatically select ROIs around the ventricles in each slice they were visible moving inferiorly to the level of the obex. The third ventricle and cerebral aqueduct were not included, and the ROIs were confirmed in three planes.

#### 2.3.3 Cerebellum

The sagittal plane was used to first identify the lateral boundaries of the cerebellar tissue. Then in the axial plane, the cerebellum was outlined based on a signal threshold and a drawing tool. The peduncles were included in the structure until the point they met the pons or medulla. The accuracy of the ROIs was confirmed again in all three planes.

#### 2.3.4 Lentiform nucleus

The lentiform nucleus was identified in the axial plane and ROIs were drawn around the structure starting superiorly on the first slice where the gray matter of the putamen was visible. The segmentation was continued inferiorly to the level of the anterior commissure. The internal capsule and external capsule were used as medial and lateral boundaries, respectively. Accuracy of the segmentation was confirmed in all three planes.

#### 2.3.5 Thalamus

In the axial plane, ROIs were manually drawn around the structure. The gray-white matter junction was used to distinguish the thalamus from the surrounding brain tissue, though this was not always completely clear on the images. Anatomic landmarks were used to ensure the accuracy of the segmentation. Ventricles were used as the superior and medial boundaries. Laterally, ROIs were not drawn past the posterior limb of the internal capsule. The segmentation was completed inferiorly until the transition to the midbrain. This ROI was then viewed and edited in all three planes to ensure anatomical accuracy.

#### 2.3.6 Corpus callosum

In the sagittal plane, the midline slice was identified using the cerebral aqueduct and the corpus callosum was outlined manually from rostrum to splenium. The segmentation was then continued laterally to the left and right for approximately the same number of slices. The ROI was then viewed in the axial plane and the slice best depicting the septum pellucidum, fornix, and foramen of Monroe was identified. Using this slice, the maximum lateral extension of the genu and splenium was determined. If needed, the border of the genu was extended at the maximum third to halfway along the frontal horns of the lateral ventricles. The borders were edited to show approximately symmetric lateral extension. Finally, the structure was viewed in the coronal plane and again in the sagittal to ensure an accurate segmentation.

#### 2.3.7 Caudate

The structure was first identified in the axial plane and ROIs were manually drawn on each slice until it was visible while moving superiorly. Inferiorly, ROIs were drawn to the level of the anterior commissure. The structure was then viewed and edited for accuracy in the sagittal and coronal planes.

### 2.4 Statistical analysis

#### 2.4.1 Dice analysis

The Sorensen-Dice and Jaccard coefficients were calculated using ImageJ (version 1.53) to compare the segmentation similarity of the brain structures between the raters (inter-rater) and between the raters' first and second segmentation attempts (intra-rater; Schneider et al., 2012). The Kruskal-Wallis test (non-parametric one-way ANOVA) was used to compare multiple groups and Dunn's multiple comparisons test was used for *post-hoc* comparisons using GraphPad Prism (version 9.5.1, GraphPad Software, San Diego, CA, USA). Statistical significance was determined by *p* < 0.05.

#### 2.4.2 ICC analysis

We assessed the agreement of the two rater teams in providing consistent segmentations by calculating the proportion of total variances in their delineated volumetric ROIs within 3D MRIs of the brain. An ICC was used to quantify the agreement between and within the previously described rater teams along with 95% confidence intervals and *p*-values for all metrics. The scores were generated using a two-way mixed effects model with k = 2 fixed raters (ICC3k) between the two different rater teams and within the rater teams themselves. In addition to the ICC metrics, we tested the required assumptions of normality, homoscedasticity, and independence. A Shapiro-Wilks test was used to assess the normality of the segmentation volumes and a Levene's test was used to assess homoscedasticity of the data. Almost all of the data had equal variances (homoscedasticity), but a significant number of the samples were not considered normally distributed. Therefore, in addition to the ICC3K metric, we also performed non-parametric correlations using Spearman rank-order correlation and Kendall's tau, which do not assume normality of the data. All calculations were performed using the Python Pingouin (version 0.5.3) and Scientific Python (Scipy, version 1.0.1) libraries.

## 3 Results

### 3.1 Juvenile and late-infantile patients

Qualitatively, differences were observed in the pattern and severity of degeneration between the late-infantile and juvenile patients. Some of these differences can be appreciated in the representative cases shown in Figure 1. Ex-vacuo dilatation of ventricles resulting from the neurodegenerative nature of the disease was present in several cases (*n* = 6 for late-infantile and *n* = 5 for juvenile) but was much more pronounced in the late-infantile patients and often led to distortion of subcortical structures, especially in the striatum and corpus callosum, making it difficult to identify and define the anatomical borders. The corpus callosum was better preserved and demarcated in juvenile patients. Overall, degeneration of cortical white matter and white matter tracts was more pronounced in the late-infantile patients. In these cases, especially, marked degeneration of the external and internal capsules made differentiation of the borders of small nuclei more challenging.

### 3.2 Dice analysis

**Figure 3:**
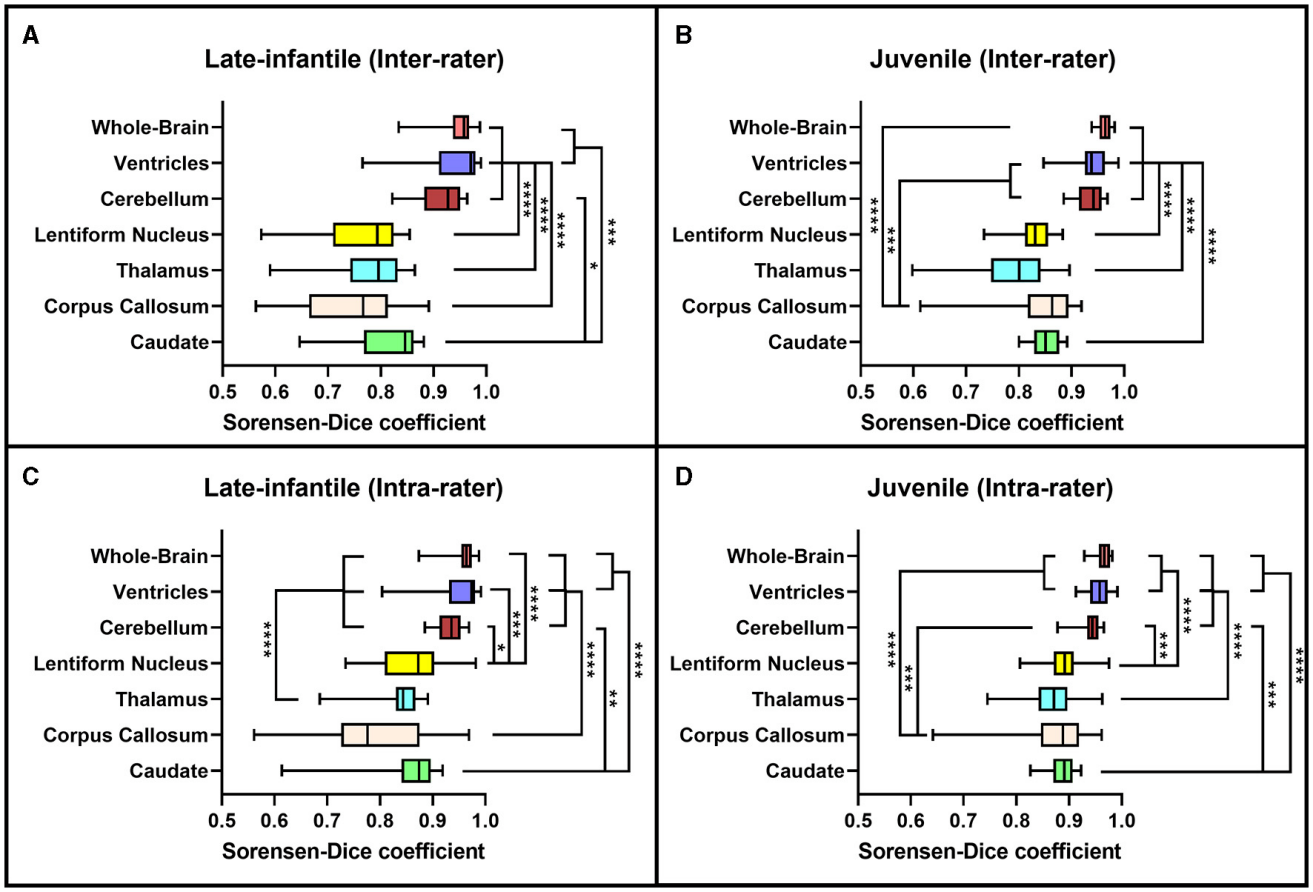
Sorensen-Dice coefficients. Box plots demonstrating the Sorensen-Dice coefficients for the inter-rater **(A, B)** and intra-rater **(C, D)** reliability of the late-infantile **(A, C)** and juvenile **(B, D)** patients. *0.01 < *p* < 0.05, **0.001 < *p* < 0.01, ***0.0001 < *p* < 0.001, ****p < 0.0001.

**Figure 4:**
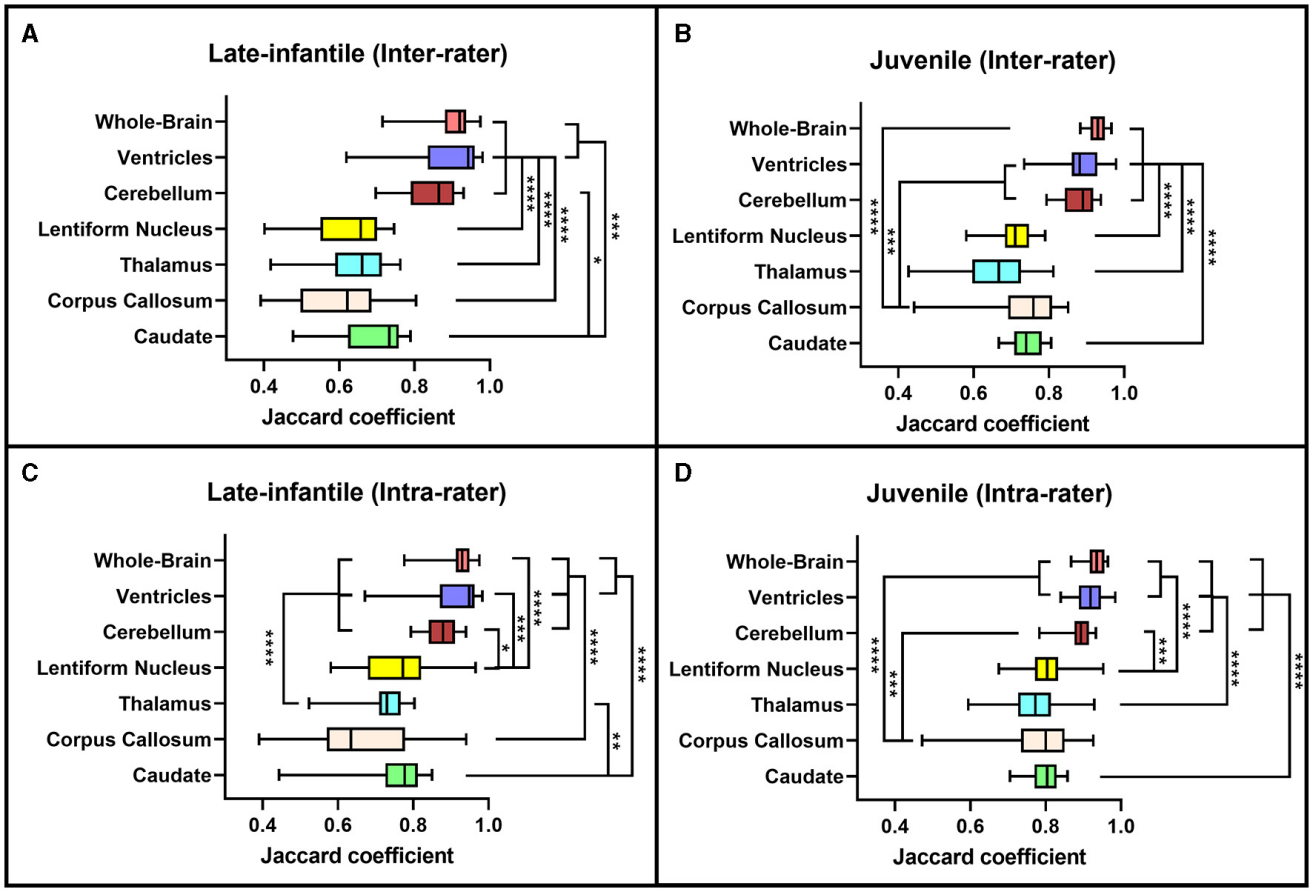
Jaccard coefficients. Box plots demonstrating the Jaccard coefficients for the inter-rater **(A, B)** and intra-rater **(C, D)** reliability of the late-infantile **(A, C)** and juvenile **(B, D)** patients. *0.01 < *p* < 0.05, **0.001 < *p* < 0.01, ***0.0001 < *p* < 0.001, ****p < 0.0001.

Based on the Sorensen-Dice and Jaccard coefficients, the inter- and intra-rater reliability of the segmentation method was significantly better for juvenile compared to late-infantile patients (*p* < 0.01). ANOVA test revealed a statistically significant difference in inter- and intra-rater reliability of structures between at least two groups (*p* < 0.0001) for both juvenile and late-infantile patients. Tukey's HSD test for multiple comparisons revealed that in general, there was a significant difference in Sorensen-Dice and Jaccard coefficients between the brain, ventricles, and cerebellum compared to the caudate, lentiform nucleus, thalamus, and corpus callosum (Figures 3, 4). The larger structures showed higher Sorensen-Dice (0.91–0.97) and Jaccard (0.85–0.94) coefficient values compared to the smaller structures (Sorensen-Dice: 0.74–0.89; Jaccard: 0.60–0.80) for both inter- and intra-rater reliability of late-infantile and juvenile patients.

### 3.3 ICC analysis

The ICC metrics were calculated for the different segmented ROIs of reconstructed brain structures to assess agreement between the two rater teams and within themselves. ICC scores can range from 0 to 1, and as is generally accepted, a score of 0.75 was used to indicate good agreement (Koo and Li, 2016). As indicated in Table 1, all the ICC values for inter- and intra-rater variability were between 0.822 and 1.000, which are in good agreement for the performance of the two rater teams. Additionally, non-parametric correlations were also calculated as some of the data violated the normality assumption for ICC. The metrics for traditional ICC (ICC3k) compared with the non-parametric correlation scores did not change the results of the agreement between the rater teams.

**Table 1:** Table of the intra-class correlation coefficients based on the measured structure volumes.

				**Inter-rater**		**Intra-rater**	
	**Structure**	**Volume (cm** ^3^ **)**	**SEM**	**ICC (95% CI)**	***p***	**ICC (95% CI)**	***p***
Late-infantile	Whole Brain	771.61	± 32.56	0.994 (0.980, 1.000)	< 0.001	0.992 (0.970, 1.000)	< 0.001
	Ventricles	81.99	± 14.79	1.000 (1.000, 1.000)	< 0.001	1.000 (1.000, 1.000)	< 0.001
	Cerebellum	87.62	± 2.98	0.990 (0.960, 1.000)	< 0.001	0.984 (0.945, 1.000)	< 0.001
	Lentiform Nucleus	9.02	± 0.40	0.822 (0.340, 0.950)	0.006	0.974 (0.905, 0.995)	< 0.001
	Thalamus	6.83	± 0.34	0.891 (0.590, 0.970)	0.001	0.956 (0.840, 0.990)	< 0.001
	Corpus Callosum	3.90	± 0.31	0.953 (0.830, 0.990)	< 0.001	0.993 (0.970, 1.000)	< 0.001
	Caudate	5.63	± 0.36	0.990 (0.960, 1.000)	< 0.001	0.985 (0.945, 1.000)	< 0.001
Juvenile	Whole Brain	851.20	± 28.44	0.998 (0.990, 1.000)	< 0.001	0.997 (0.990, 1.000)	< 0.001
	Ventricles	40.88	± 7.98	1.000 (1.000, 1.000)	< 0.001	1.000 (1.000, 1.000)	< 0.001
	Cerebellum	102.67	± 2.31	0.978 (0.930, 0.990)	< 0.001	0.982 (0.945, 0.995)	< 0.001
	Lentiform Nucleus	9.49	± 0.26	0.962 (0.880, 0.990)	< 0.001	0.968 (0.900, 0.990)	< 0.001
	Thalamus	9.55	± 0.34	0.895 (0.670, 0.970)	< 0.001	0.924 (0.760, 0.975)	< 0.001
	Corpus Callosum	6.60	± 0.29	0.966 (0.900, 0.990)	< 0.001	0.959 (0.875, 0.985)	< 0.001
	Caudate	5.47	± 0.18	0.985 (0.950, 1.000)	< 0.001	0.985 (0.955, 0.995)	< 0.001

## 4 Discussion

Segmentation of important whole and specific brain structures can be used to determine quantitative brain MRI volumetrics at different stages of disease progression in patients with GM1. This provides a useful, non-invasive, objective imaging biomarker to understand natural disease progression and may be used to evaluate treatment response for future trials. Current approaches for automated segmentation of brain structures do not generalize well to patients with GM1. Atrophy and parenchymal signal changes resulting from neurodegenerative processes in these patients preclude accurate image registration and normalization, which are critical steps in automatic segmentation pipelines (Nestrasil et al., 2018). Manual segmentation, although time consuming and prone to both inter- and intra-rater variability as seen in other studies (Yu et al., 2010; Hashempour et al., 2019; Phan et al., 2018; John et al., 2006; Entis et al., 2012), remains at present the only realistic method to quantitatively track brain structure volumes in these patients. Here we introduce a standardized pipeline to manually segment brain structures on MRI in a cohort of patients with type II GM1 and assess the inter- and intra-rater reliability of this segmentation method. Our results provide specific guidelines for manual segmentation of regions known to be implicated in GM1 patients while considering the substantial variability in myelination and brain development at different stages of the disease. The segmentation protocol presented herein can be performed using other software tools with appropriate editing functionalities.

Both the inter- and intra-rater reliability assessment demonstrated our segmentation methodology to be efficacious in generating reproducible brain volumetric quantifications. The results were noted to be better for the juvenile compared to the late-infantile patients. Additionally, there was higher spatial overlap both within and between rater teams for whole brain, ventricle and cerebellar volumes compared to volumes of deep nuclei and the corpus callosum; this was particularly pronounced for the late-infantile subtype. These differences may reflect the rate of progression of disease in these two subtypes with early and more rapid progression of anatomic changes including brain atrophy and hypomyelination in the late-infantile subtype compared to the milder progression reported in the juvenile subtype (Regier et al., 2016a; Nestrasil et al., 2018; Rha et al., 2021). Basal ganglia pathology is also often documented in GM1 gangliosidosis. For example, both Nestrasil et al. and Regier et al. described marked atrophy of the basal ganglia in GMI patients and Kasma et al. reported accumulation of GM1 ganglioside in the caudate and putamen (Regier et al., 2016a; Nestrasil et al., 2018; Kasama and Taketomi, 1986). Moreover, abnormal findings within the thalamus have been reported in the infantile and late-infantile subtypes of GM1 gangliosidosis, but not in the juvenile subtype (Rha et al., 2021). These differences in neurodegenerative processes and resultant signal intensity changes may contribute to poor delineation of boundaries for accurate segmentation in the late-infantile subtype compared to the juvenile subtype and for smaller structures compared to the larger cerebral and cerebellar volumes. Additionally, pixel intensity was used to help automate segmentation of the cerebrum, cerebellum, and ventricles while segmentations of the other structures were entirely manual.

The natural history of GM1 gangliosidosis remains poorly understood due to a paucity of research evaluating the longitudinal progression of central nervous system changes and their correlation with clinical findings. The majority of published studies of neuroimaging findings in these patients have been descriptive case reports (De Grandis et al., 2009; Erol et al., 2006). There have been only two studies utilizing quantitative brain MRI volumetric analyses to quantify and compare between infantile and juvenile forms of the disease (Nestrasil et al., 2018; Regier et al., 2016b). In a longitudinal study that included infantile, late-infantile, and juvenile patients with both GM1 and GM2 gangliosidoses, Nestrasil et al. reported significantly decreased brain volumes across multiple regions (Nestrasil et al., 2018). Regier et al. demonstrated that late-infantile patients with GM1 had more severe progressive atrophy of the cerebrum, cerebellum, and hippocampus compared to the juvenile subtype (Regier et al., 2016b). Results in both of these studies correlated with clinical symptoms. These studies highlight the ongoing need for objective biomarkers to evaluate disease burden, progression, and treatment responses in these patients. Finally, although designed for this specific patient population, the methods presented here may be generalizable for studying other similar neurodegenerative diseases.

There are several limitations in our investigation. Firstly, we report on a small cohort of patients with type II GMI gangliosidosis, which is unavoidable in this rare disease. Conceivably, a larger sample size may introduce additional variations in neuroanatomy and these variations may alter the data reliability. While replication in a larger sample may be helpful, high inter- and intra-rater reliability was achieved in our study despite the small sample size suggesting robustness of our segmentation approach. Secondly, our manual segmentation pipeline demonstrated more inter- and intra-rater variability for segmentation of small structures compared to larger structures. In future works, the proposed protocol could be used to generate training data for artificial intelligence-based methods to automatically delineate anatomic boundaries using deep learning techniques. More detailed guidelines may be required for improving the quality of the manual segmentation.

## 5 Conclusions

With the potential emergence of novel therapies for GM1 patients, such as the clinical trials where GM1 patients are being treated with gene therapy, brain volumetrics is one of the neuroimaging biomarkers of interest that can potentially track the state of the disease; hence, accurate and reproducible volumetric assessments that provide early evidence of response or progression are essential. The approach described in this study will provide greater accuracy and reproducibility for neuromorphological studies in GM1 patients as well as in other neurodegenerative disorders and may help in further expanding our understanding of the natural history of this disease.

## Data Availability

Datasets can be made available to investigators upon reasonable request. Requests to access these datasets should be directed to: mohammed.shazeeb@umassmed.edu.
